# Effect of time-varying adherence to non-pharmaceutical interventions on the occurrence of multiple epidemic waves: A modeling study

**DOI:** 10.3389/fpubh.2022.1087683

**Published:** 2022-12-20

**Authors:** Joel-Pascal Ntwali N'konzi, Chidozie Williams Chukwu, Farai Nyabadza

**Affiliations:** ^1^African Institute for Mathematical Sciences, Kigali, Rwanda; ^2^Maxwell Institute for Mathematical Sciences, University of Edinburgh, Heriot-Watt University, Edinburgh, United Kingdom; ^3^Department of Mathematics and Applied Mathematics, University of Johannesburg, Johannesburg, South Africa

**Keywords:** SEIR model, non-pharmaceutical interventions, time-varying adherence, epidemic waves, numerical simulations

## Abstract

Non-pharmaceutical interventions (NPIs) play a central role in infectious disease outbreak response and control. Their usefulness cannot be overstated, especially during the early phases of a new epidemic when vaccines and effective treatments are not available yet. These interventions can be very effective in curtailing the spread of infectious diseases when adequately implemented and sufficiently adopted by the public. However, NPIs can be very disruptive, and the socioeconomic and cultural hardships that come with their implementation interfere with both the ability and willingness of affected populations to adopt such interventions. This can lead to reduced and unsteady adherence to NPIs, making disease control more challenging to achieve. Deciphering this complex interaction between disease dynamics, NPI stringency, and NPI adoption would play a critical role in informing disease control strategies. In this work, we formulate a general-purpose model that integrates government-imposed control measures and public adherence into a deterministic compartmental epidemic model and study its properties. By combining imitation dynamics and the health belief model to encode the unsteady nature of NPI adherence, we investigate how temporal variations in NPI adherence levels affect the dynamics and control of infectious diseases. Among the results, we note the occurrence of multiple epidemic waves as a result of temporal variations in NPI adherence and a trade-off between the stringency of control measures and adherence. Additionally, our results suggest that interventions that aim at increasing public adherence to NPIs are more beneficial than implementing more stringent measures. Our findings highlight the necessity of taking the socioeconomic and cultural realities of affected populations into account when devising public health interventions.

## 1. Introduction

An important aspect of the control of infectious diseases is the use of non-pharmaceutical interventions (NPIs) such as social distancing, mask-wearing, school closures, mass gathering bans, and lockdowns that aim to change key aspects of human behavior to reduce transmission rates. Their usefulness cannot be overstated, especially during the early phases of a new epidemic when vaccines are not available yet. The still ongoing COVID-19 pandemic has underscored the value of these interventions even beyond the early phases of an epidemic. Various studies have shown, for instance, the necessity of keeping NPIs in place during the vaccine rollout in 2021, a year after the start of the pandemic (1, 2). The literature on the usefulness of NPIs and their effects on the transmission dynamics of COVID-19 is vast (3–9).

However, as the COVID-19 pandemic has shown, disease control measures can come with unprecedented damages to economies and overall well-being that usually interfere with the willingness and capabilities of the public to adopt recommended health behaviors. This can lead to reduced and unsteady adherence to NPIs, making disease control more challenging to achieve. Additionally, the COVID-19 pandemic has highlighted the fact that faced with the same public health threat, countries will implement interventions of varied stringency and the level of public adherence to these interventions will vary widely between regions and over time (10–15). This has consequences on the effectiveness of NPIs over time. In particular, the study by Ge et al. (16) has highlighted the fluctuating nature of NPI performance over time at different geographical scales. These observations are not particular to the COVID-19 pandemic. The works by Matthews Pillemer et al. (17) and Wang et al. (18) showed, for instance, that similar patterns had been observed during the 2002-03 SARS outbreak in four different regions around the world and the 2013 H7N9 outbreak in China, respectively.

Many studies have investigated the predictors of adherence to NPIs during public health emergencies and causes of variations in adherence levels over time, especially during the COVID-19 pandemic (13, 17, 19–22). The most commonly identified factors that affect compliance with NPIs are risk perception, trust or lack of trust in public health authorities, misinformation, economic hardship, and the sociocultural realities of affected populations. NPI adherence patterns can significantly impact the effectiveness of disease control strategies and interact largely with disease dynamics over time. Factors like risk perception and perceived economic hardship due to NPI adherence are subject to temporal change as the disease unfolds resulting in a complex interaction between disease dynamics, the stringency of NPIs, and NPI adherence.

Untangling the feedback loop between human behavior and the spread of infectious diseases has caught the attention of many over the last two decades due to its importance for epidemic preparedness and control and this has led to what is now called *behavioral epidemiology of infectious diseases* (23–26). Though the pre-COVID-19 literature is vast, we have not come across many studies related to modeling the interplay between NPI stringency, NPI adherence, and the dynamics of infectious diseases. Much progress has been made since the beginning of the COVID-19 pandemic, however. The studies by Acuña-Zegarra et al. (27) and Iyaniwura et al. (28) both investigated the impact of adherence to NPIs on the dynamics of COVID-19 using an SEIR-like model. In particular, they both divide the population into 2 groups: individuals who adhere to all the NPIs are put in one group, and those who do not adhere in the other group. To capture the time-varying nature of NPI adherence, the two models allow flows between the 2 groups, though the model by Acuña-Zegarra et al. (27) incorporates only the movement from the adherents to the non-adherents group. However, though their modeling framework allows highlighting the effect of NPI adherence on disease dynamics, it is agnostic on the drivers of changes in NPI adherence status. Additionally, since most individuals only partially adhere to NPIs, it does not seem sensible to divide the population into adherents and non-adherents. Finally, their models do not capture the interaction between NPI intensity and NPI adherence, nor do they allow to answer the question of how disease dynamics interact with either of the two.

Other modeling studies on NPIs focused on assessing the effectiveness of NPIs or their usefulness in specific contexts (29–31). Though these models have played a major role in shedding light on the need for sustained adherence to NPIs during the COVID-19 pandemic, the authors studied the effect of NPI adherence on disease dynamics by simulating the models under different *fixed* NPI adherence scenarios, and thus failing to account for the impacts of temporal variations in NPI adherence on disease dynamics.

In this study, we aim to fill in this gap. We modify the classical deterministic Susceptible-Exposed-Infected-Recovered (SEIR) model to capture both the effect of disease control measures and the feedback loop between NPI adherence and disease dynamics. We use the model to investigate the effect of temporal variations in NPI adherence levels on the spread of infectious diseases. We use imitation dynamics and build upon the health belief model to encode the dynamics of the public level of adherence to NPIs. We do not take into account the intricacies of specific diseases such as the spread of different variants or strains and the availability of vaccines for sake of generality. The approach we use for modeling temporal variations in NPI adherence is similar to the one adopted in Jentsch et al. (32).

The rest of this paper is organized as follows. In Section 2 we present the mathematical model used in this study. Section 3 contains our results. In particular, we derive an expression for the basic reproduction number ($R_{0}$) and study the stability properties of the equilibrium solutions of the model. Furthermore, we perform a scenario analysis to investigate the effect of both the stringency of NPIs and temporal variations in the level of adherence on the disease dynamics. Lastly, in Section 4 we provide a discussion of our results.

## 2. Methods

### 2.1. Baseline model

We use a deterministic SEIR model that divides the population into four distinct compartments based on the respective disease state of individuals. The *Susceptible* (S) compartment contains individuals who can but have not yet been infected by the disease. Individuals in the *Exposed* (E) have been infected by the disease but cannot spread it, whereas those in the *Infected* (I) compartment can transmit the disease. The *Recovered* (R) compartment contains individuals who have recovered from the disease. Our model takes into account births and both natural and disease-induced deaths. For simplicity, we do not take reinfections into account. That is, we assume that individuals who recover from the disease become immune over the course of the epidemic. This is the case for diseases like measles, for example. This leads to the following system of differential equations:


(1a) $$\begin{array}{l} \frac{\text{d}S}{\text{d}t}=\pi -\lambda \left(t\right)+\mu S, \end{array}$$

(1b) $$\begin{array}{l} \frac{\text{d}E}{\text{d}t}=\lambda \left(t\right)S-\left(\kappa +\mu \right)E, \end{array}$$

(1c) $$\begin{array}{l} \frac{\text{d}I}{\text{d}t}=\kappa E-\left(\gamma +\delta +\mu \right)I, \end{array}$$

(1d) $$\begin{array}{l} \frac{\text{d}R}{\text{d}t}=\gamma I-\mu R, \end{array}$$

with nonnegative initial conditions *S*(0) = *S*_0_, *E*(0) = *E*_0_, *I*(0) = *I*_0_, *R*(0) = *R*_0_, and *x*(0) = *x*(0). The disease state variables *S, E, I*, and *R* represent fractions of the population in the corresponding compartment, and model parameters are as described in Table 1 below.

**Table 1:** Description of state variables and model parameters.

**Variables**	**Description**
*S*	Fraction of susceptible individuals
*E*	Fraction of exposed individuals
*I*	Fraction of infectious individuals (prevalence rate)
*R*	Fraction of recovered individuals
*x*	Level of adherence to recommended control measures
**Parameters**	**Description**
π	Birth/recruitment rate
β_0_	Effective contact rate in the absence of disease control measures
μ	Natural death rate
1/κ	Mean latent period
γ	Recovery rate of infected individuals
δ	Disease-induced per capita death rate
*α*	Disease control strength
*r*_*i*_	Overall perceived susceptibility
*m*	Overall perceived severity of the disease
*r*_*c*_	Overall perceived frustration with control measures
*k*	Overall sensitivity to changes in disease prevalence
*ω*	*r*_*i*_*m*/*r*_*c*_
*K*	*k*/*r*_*c*_

In the classical SEIR model, non-pharmaceutical interventions are not explicitly taken into consideration, thus the *force of infection*, λ(*t*), is given by


(2) $$\begin{array}{l} \text{λ}\left(t\right)=\beta_{0}I\left(t\right), \end{array}$$

where β_0_ is the *effective contact rate* or *transmission rate* in the absence of control measures and *I*(*t*) is the prevalence rate of the disease in the population at time *t*, i.e., the ratio of the number of infected individuals to the total population count.

### 2.2. Incorporating control measures

To incorporate the effect of NPIs, we assume that the stringency of interventions is proportional to the prevalence rate of the disease and that they are geared toward reducing the transmission rate of the disease. Hence, we use a prevalence-dependent contact rate and modify the force of infection in Equation (2) to obtain:


(3) $$\begin{array}{l} \text{λ}\left(t\right)=\frac{\beta_{0}I\left(t\right)}{1+\alpha I\left(t\right)}, \end{array}$$

where *α* is a positive, constant parameter that measures the stringency of government policy responses to curb the epidemic. A probably more insightful way of thinking about the parameter *α* is that the fraction 1/*α* defines the prevalence rate that has to be reached within the population for the policymakers to implement NPIs that aim to reduce the transmission rate by half. Hence, high values of *α* mean that decision-makers have a stringent policy approach in responding to the epidemic whereas lower values correspond to instances of a more hands-off policy approach. It is worth noting that the use of prevalence-dependent contact rates to model behavioral responses to disease outbreaks as in Equation (3) can be traced back to the work of Capasso and Serio (33) and was used by Gros et al. (34) to model short-term control strategies for the SARS-CoV-2 virus.

An important limitation of the force of infection in Equation (3) is that it implicitly assumes that individuals' compliance with disease control measures is constant over time. However, as shown earlier, this is not realistic in many instances.

### 2.3. Modeling the dynamics of NPI adherence level

To model temporal variations in adherence levels to NPIs, we introduce an additional variable, *x*(*t*), that measures the *level of adherence* to disease control measures at time *t*. We modify the force of infection in Equation (3) to obtain:


(4) $$\begin{array}{l} \lambda \left(t\right)=\frac{\beta_{0}I}{1+\alpha Ix\left(t\right)}. \end{array}$$

We assume that *x*(*t*) is an average over the population. Building upon the health belief model (35) and using imitation dynamics, as in the work by Bauch (36) on vaccinating behavior, we model the dynamics of the adherence level by


(5) $$\begin{array}{l} \frac{\text{d}x}{\text{d}t}=kx\left(1-x\right)\left(-r_{c}+r_{i}mI\right), \end{array}$$

where *r*_*c*_ represents the *perceived frustration* with NPIs due to the socioeconomic, cultural, and emotional damages and inconveniences associated with NPI adoption; *r*_*i*_ and *m* represent the *perceived susceptibility* to the disease and the *perceived severity* of the disease, respectively; and *k* measures the *public responsiveness* to changes in disease prevalence. Defining *K* = *k*/*r*_*c*_ and *ω* = *r*_*i*_*m*/*r*_*c*_ as in Bauch (36), we then rewrite Equation (5) as:


(6) $$\begin{array}{l} \frac{\text{d}x}{\text{d}t}=Kx\left(1-x\right)\left(-1+\omega I\right), \end{array}$$

where $I=\frac{1}{\omega }$ is a threshold prevalence rate below which the adherence level would be decreasing. Hence, the two threshold prevalence rates *I* = 1/*α* and *I* = 1/*ω* are loose measures of tolerance for the disease at the decision-makers level and public level, respectively.

## 3. Results

In this section, we derive a mathematical expression for the basic reproduction number, $R_{0}$, and study the stability properties of both disease-free and endemic equilibrium solutions in Section 3.1. In particular, we show that the model has two disease-free equilibria, of which one is always unstable and the other is globally asymptotically stable when $R_{0}<1$ and unstable otherwise. When $R_{0}>1$, the model has three endemic equilibrium solutions that exchange stability as $R_{0}$ is varied. These analytical results are followed by extensive numerical simulations in Section 3.2.

### 3.1. Equilibria and basic reproduction number

Our model has two disease-free equilibria given by


(7) $$\begin{array}{l} E_{1}=\left(S_{1},E_{1},I_{1},R_{1},x_{1}\right)=\left(\frac{\pi }{\mu },0,0,0,0\right), \end{array}$$

and


(8) $$\begin{array}{l} E_{2}=\left(S_{2},E_{2},I_{2},R_{2},x_{2}\right)=\left(\frac{\pi }{\mu },0,0,0,1\right), \end{array}$$

corresponding to the case where there is no disease and no adherence to NPIs for $E_{1}$, and the situation where the disease is absent but the population fully complies with prevention measures $E_{2}$.

The basic reproduction number, $R_{0}$, for this model is given by


(9) $$\begin{array}{l} R_{0}=\frac{\beta_{0}\kappa \pi }{\chi \mu }, \end{array}$$

with *χ* *=* (*κ+μ*)(*γ+δ+μ*).

The disease-free equilibrium point $E_{1}$ is globally asymptotically stable when $R_{0}<1$ and unstable when $R_{0}>1$, whereas $E_{2}$ is always unstable (proof in Section 1 of the Supplementary material). This encapsulates the fact that when there is no disease, individuals have no incentive to adhere to NPIs, especially because the cost of adopting preventive measures, no matter how small it might be, would still be higher than the perceived risk of acquiring the disease when the disease is absent.

Additionally, the model has rich dynamics with three endemic equilibria given by

**(a)** $E_{3}\equiv \left(S^{*},E^{*},I^{*},R^{*},x^{*}\right)$, with

(10) $$\begin{array}{l} \begin{array}{l} S^{*}=\frac{\chi }{\beta \kappa },\\ E^{*}=\frac{\mu \left(\gamma +\delta +\mu \right)}{\kappa \beta }\left(R_{0}-1\right),\\ I^{*}=\frac{\mu }{\beta }\left(R_{0}-1\right),\\ R^{*}=\frac{\gamma }{\beta }\left(R_{0}-1\right),\\ x^{*}=0, \end{array} \end{array}$$

exists when $R_{0}>1$.

**(b)** $E_{4}\equiv \left(S^{*},E^{*},I^{*},R^{*},x^{*}\right)$, with

(11) $$\begin{array}{l} \begin{array}{c} S^{*}=\frac{1}{\mu }\left(\pi -\frac{\chi }{\kappa \omega }\right)=\frac{\pi }{R_{0}}\left(R_{0}-\frac{\beta }{\mu \omega }\right),\\ E^{*}=\frac{\gamma +\delta +\mu }{\kappa \omega },\\ I^{*}=\frac{1}{\omega },\\ R^{*}=\frac{\gamma }{\mu \omega },\\ x^{*}=\frac{1}{\alpha }\left[R_{0}-\left(1+\frac{\beta }{\mu \omega }\right)\right], \end{array} \end{array}$$

exists when


(12) $$\begin{array}{l} R_{0}>1+\frac{\beta }{\mu \omega }, \end{array}$$

and

**(c)** $E_{5}\equiv \left(S^{*},E^{*},I^{*},R^{*},x^{*}\right)$, with

(13) $$\begin{array}{l} \begin{array}{c} S^{*}=\frac{\alpha \pi \kappa +\chi }{\kappa \left(\beta +\alpha \mu \right)},\\ E^{*}=\frac{\mu \left(\gamma +\delta +\mu \right)}{\kappa \left(\beta +\alpha \mu \right)}\left(R_{0}-1\right),\\ I^{*}=\frac{\mu }{\beta +\alpha \mu }\left(R_{0}-1\right),\\ R^{*}=\frac{\gamma }{\beta +\alpha \mu }\left(R_{0}-1\right),\\ x^{*}=1, \end{array} \end{array}$$

exists whenever $R_{0}>1$.

** Theorem 1 (Stability of $E_{3}$)**. The endemic equilibrium point $E_{3}$ is locally asymptotically stable when


(14) $$\begin{array}{l} 1<R_{0}<1+\frac{\beta }{\mu \omega }, \end{array}$$

and unstable otherwise.

** Theorem 2 (Stability of $E_{5}$)**. The endemic equilibrium point $E_{5}$ is locally asymptotically stable when


(15) $$\begin{array}{l} R_{0}>1+\frac{\beta }{\mu \omega }+\frac{\alpha }{\omega }\; \end{array}$$

(16) $$\begin{array}{l} \text{and }R_{0}>\frac{1}{\mu }\left(\frac{\chi }{\xi_{0}}-1\right),\; \end{array}$$

where χ = (γ+δ+μ)(κ+μ) and ξ_0_ = γ+δ+κ+2μ. Moreover, if we assume the latency period is at least 1 day so that κ ≤ 1, then (Equation 15) subsumes (Equation 16). The endemic equilibrium $E_{5}$ is unstable when $R_{0}<1+\frac{\beta }{\mu \omega }+\frac{\alpha }{\omega }$.

Theorem 1 shows that for diseases that eventually spread within a population, the higher *ω* is, the more likely the public will keep some level of adherence to NPIs when the disease becomes endemic. On the other hand, Theorem 2 shows that the likelihood of having full adherence to NPIs during the endemic stage is both proportional to *ω* and inversely proportional to the stringency of control measures, *α*. This highlights a trade-off between NPI stringency and adherence. (Proofs can be found in Section 2 of the Supplementary material).

### 3.2. Scenario analysis

We simulate the modified SEIR model given by Equations (1), (3), and (6) in different scenarios to better understand the effect of temporal variations in NPI adherence levels on disease dynamics. We keep all the epidemiological parameters fixed and vary only the values of *α*, the stringency of control measures, and *ω*, with 1/*ω* measuring the tolerance of the disease in the public. Parameter values used in simulations are given in Table 2.

**Table 2:** Parameter values used in simulations.

**Parameter**	**Figures 1–5**	**Figure 6**
π	0.000002	0.0017
μ	0.000002	0.0017
β_0_	1.1	0.6
κ	1/6	0.2
γ	0.125	0.2
δ	0.001	0.004
*K*	0.4	0.3

#### 3.2.1. Baseline scenario

**Figure 1:**
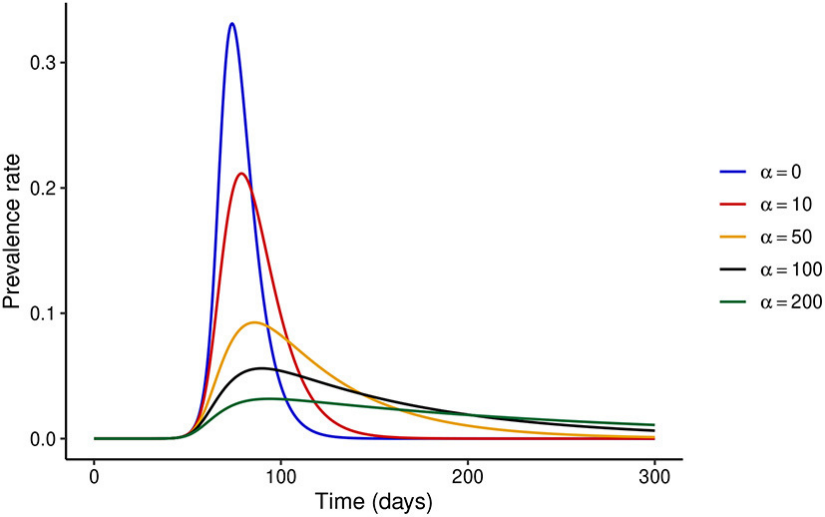
Effect of increasing NPI stringency when the adherence level to NPIs is constant over time. It is clear that under this assumption, substantial increases in NPI stringency result in a considerable decrease in the prevalence at the peak of the epidemic.

First, we consider the hypothetical situation where the NPI adherence level is fixed over time, i.e., *x*(*t*) = *x*. When *x* = 0 or *α* = 0, the model reduces to the classical SEIR model without the effect of disease control interventions. It is easy to see that when the adherence level is constant over time, one can write *x*(*t*) = 1 without any loss of generality. We investigate the dynamics of the disease when the adherence level is fixed at *x* = 1 for different values of *α*. Figure 1 shows that increasing the stringency of NPIs can result in a substantial decrease in the prevalence at the peak of the epidemic. It is important to note that the model predicts that the epidemic curve can be flattened for high values of *α*, and the ultimate conclusion would be that the higher the stringency of NPIs, the better. However, this might not hold when temporal variations in NPI adherence are considered, as we show below.

#### 3.2.2. Time-varying adherence level

##### 3.2.2.1. Fixed NPI stringency

**Figure 2:**
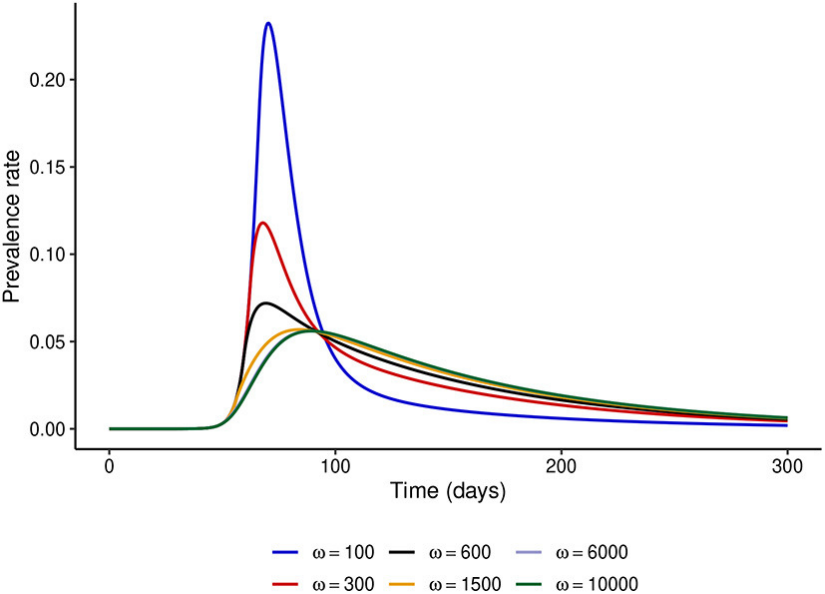
Effect of increasing *ω* at fixed *α* = 100. This figure shows that when the level of public tolerance for the disease is high, the prevalence at the peak of the epidemic can be much higher than what might be expected under the fixed NPI adherence level assumption.

First, we fix the stringency of NPIs to *α* = 100 and investigate the effect of varying *ω*. Figure 2 shows that if *ω* = 100, i.e., the risk perceived by the public exceeds the barriers to NPI adherence only when disease prevalence is at least 1%, then the peak prevalence of the epidemic would exceed 20%, whereas it was lower than 10% even for mildly stringent NPIs (*α* = 50) when the NPI adherence level was assumed constant throughout the epidemic. However, if the public perceives a much higher risk from the disease (*ω* ≥ 6,000), the level of adherence to control measures would be much higher and the peak prevalence can be reduced to around 6%, which is much closer to what was observed in Figure 1. It is noteworthy that even under perfect adherence to NPIs, the prevalence at peak cannot be further reduced without increasing the stringency of control measures.

##### 3.2.2.2. Fixed public tolerance for the disease

**Figure 3:**
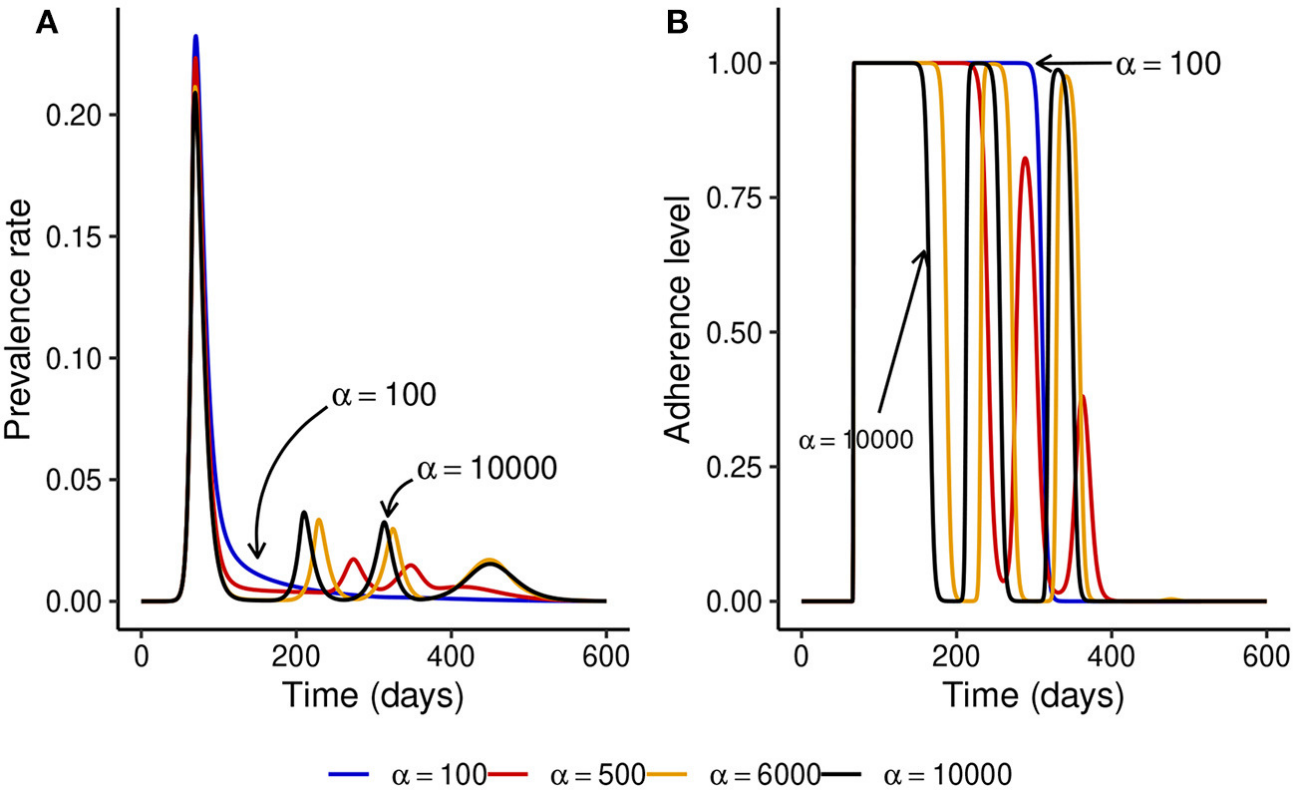
Effect of increasing the stringency of NPIs, *α*, when the level of adherence to NPIs is time-varying and *ω* = 100 is fixed. This shows that if the public tolerance for the disease is not sufficiently low, increasing NPI stringency results only in a marginal decrease in the peak prevalence and can induce the occurrence of multiple epidemic waves. This is in contrast with what is observed in Figure 1 when the NPI adherence level is constant over time. **(A)** The prevalence rate of the disease. **(B)** Time-varying adherence level to NPIs.

We observed that under the fixed adherence assumption, i.e., *x*(*t*) = 1 for all *t*, a substantial increase in NPI stringency would result in a substantial decrease in the peak prevalence for relatively low values of *α*. However, this might not be the case when the NPI adherence level, *x*(*t*), is time-varying. To see what might happen in this case, we fix *ω* = 100 and simulate the model for different values of *α*. Figure 3 shows that increasing the NPI stringency, *α*, does not result in a substantial decrease in the peak prevalence when the public tolerance for the disease is not sufficiently low. This is in contrast with what is observed in Figure 1 when the NPI adherence level is constant over time. Moreover, for considerably high values of *α*, the level of adherence to NPIs fluctuates over time due to trade-offs between NPI stringency and adherence, leading to the occurrence of multiple epidemic waves. This seemingly counter-intuitive observation might be justified by the quite high public tolerance of the disease when *ω* = 100 as the NPI adherence level increases only when the disease prevalence is at least 1%. This might happen either because the perceived risk for the disease is low or because the perceived frustration with NPIs is much higher than the perceived risk. This shows that not much can be achieved by imposing very stringent control measures if the capacity and willingness of the public to adopt such measures are not increased.

##### 3.2.2.3. Striking the right balance between NPI stringency and adherence

**Figure 4:**
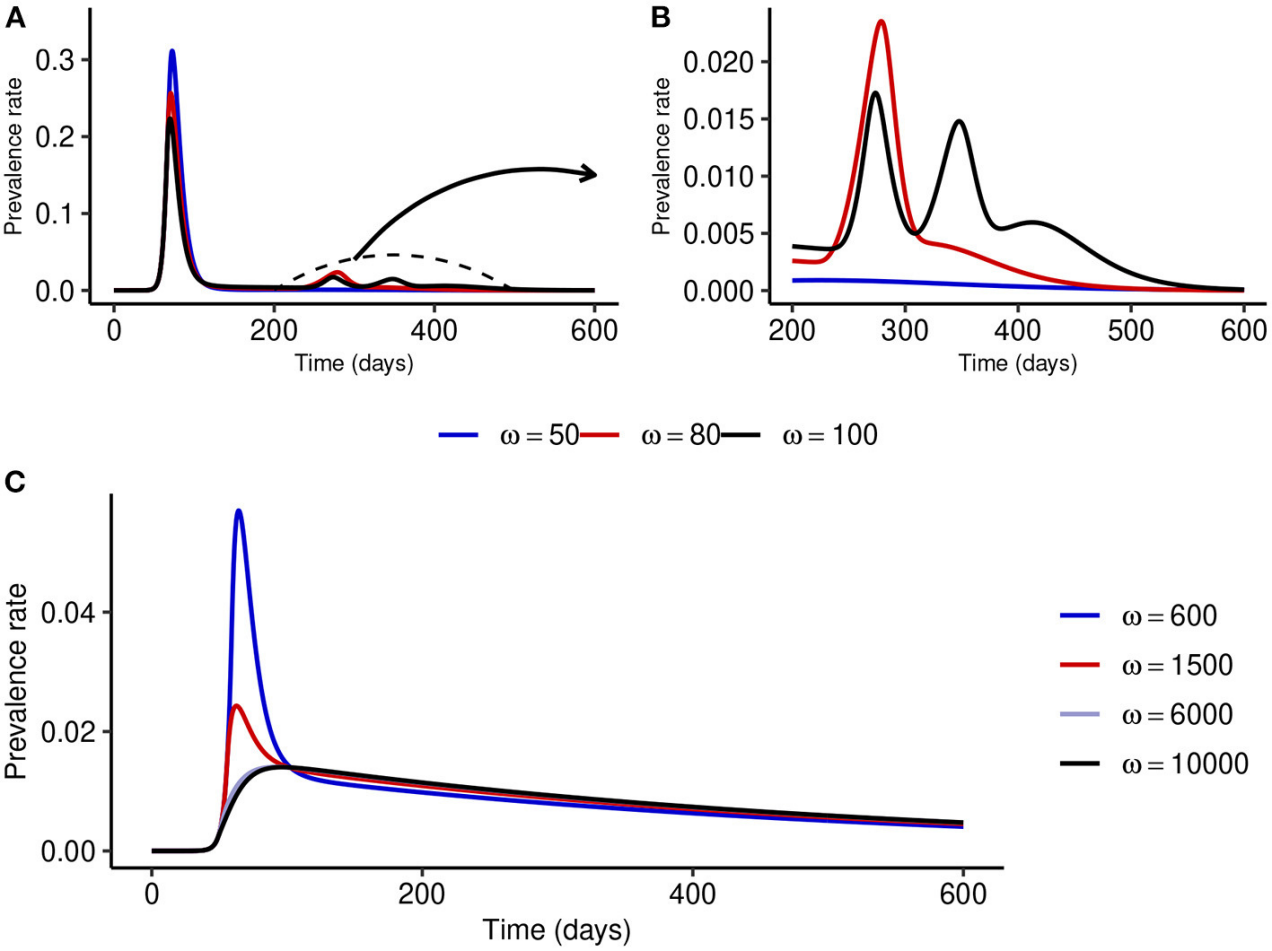
Effect of increasing *ω* at fixed medium-level NPI stringency (*α* = 500). **(A, B)** Show that multiple epidemic waves can occur when *ω* is not high enough. Both the number of secondary waves and the prevalence at each secondary peak depend on the stringency of NPIs, *α*, and the public tolerance for the disease, 1/*ω*. **(C)** Shows that the epidemic curve can be flattened for considerably high values of *ω*.

To understand what might happen when NPI stringency is high but not too high, we fix NPI stringency at *α* = 500 and simulate the model for different values of *ω*. Figures 4A, B show that for small values of *ω*, multiple epidemic waves can occur due to the trade-offs between NPI stringency and adherence. However, as *ω* is increased, the effectiveness of NPIs increases and the epidemic curve can be flattened (see Figure 4C). It is important to note that the peak prevalence for the flattened epidemic curve (*ω* = 6,000) is around 1.4%, which is much lower compared to the 31.1% when *ω* = 50 and lower than the 1.7% prevalence peak for the second wave when *ω* = 100. This suggests that disease control can be much more effective with reasonably stringent NPIs if the willingness and capacity of the public to adopt such measures is increased.

##### 3.2.2.4. What matters most: Increasing NPI stringency or adherence?

**Figure 5:**
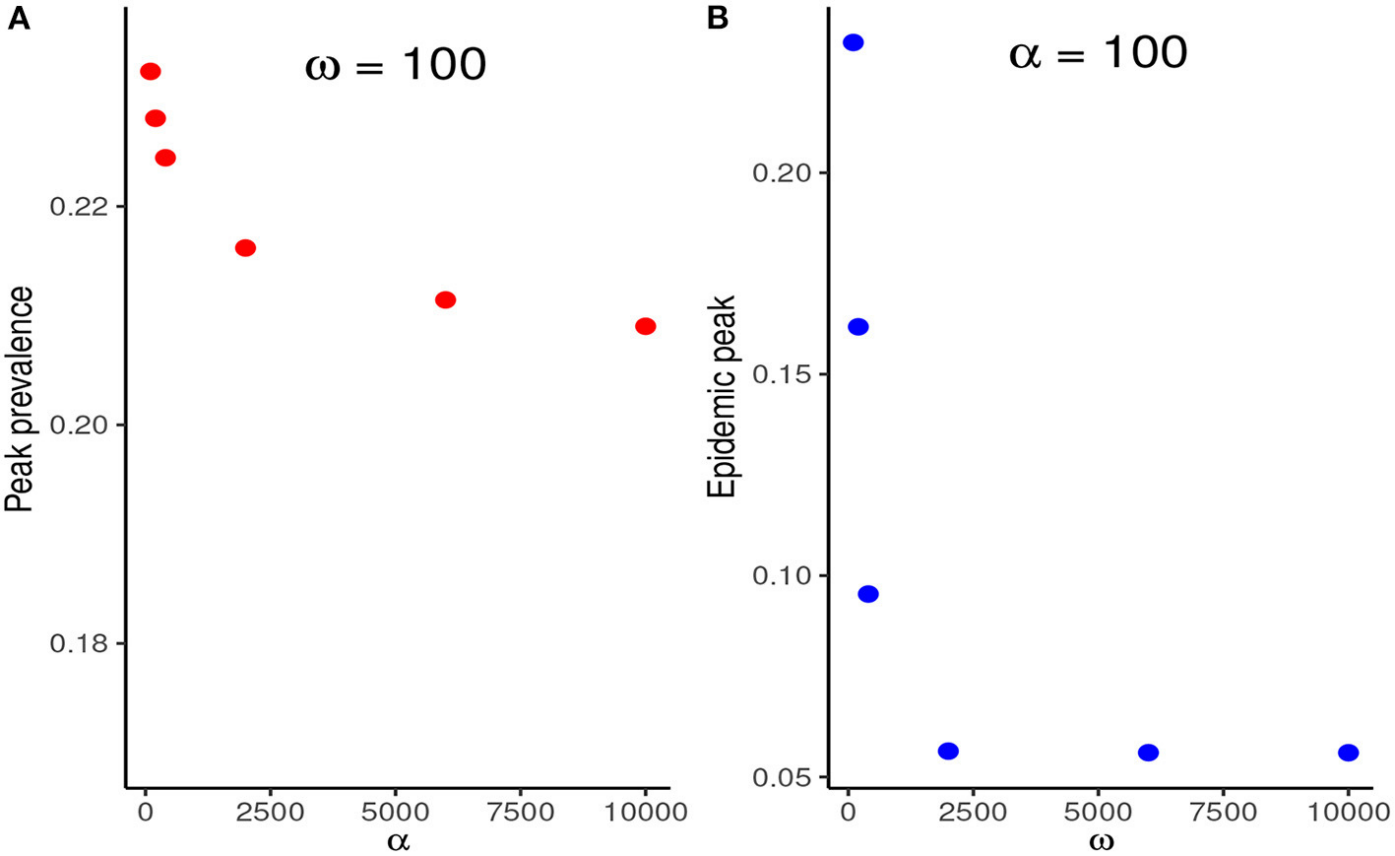
**(A)** Reduction in the peak prevalence when *α* is increased at fixed *ω* contrasted with the reduction when *ω* is increased at fixed *ω*. **(B)** Increasing *ω*, and thus the NPI adherence level, therefore, results in much higher marginal benefits than increasing the stringency of NPIs at fixed *ω*.

We compare the relative reduction in the prevalence at the first peak when *α* is increased at fixed *ω* and when *ω* is increased at fixed *α*. Figure 5 shows that increasing *ω*, and thus the adherence level to NPIs, results in a much more substantial reduction in the peak prevalence than increasing NPI stringency, *α*, at fixed *ω*.

##### 3.2.2.5. Equilibrium solutions

**Figure 6:**
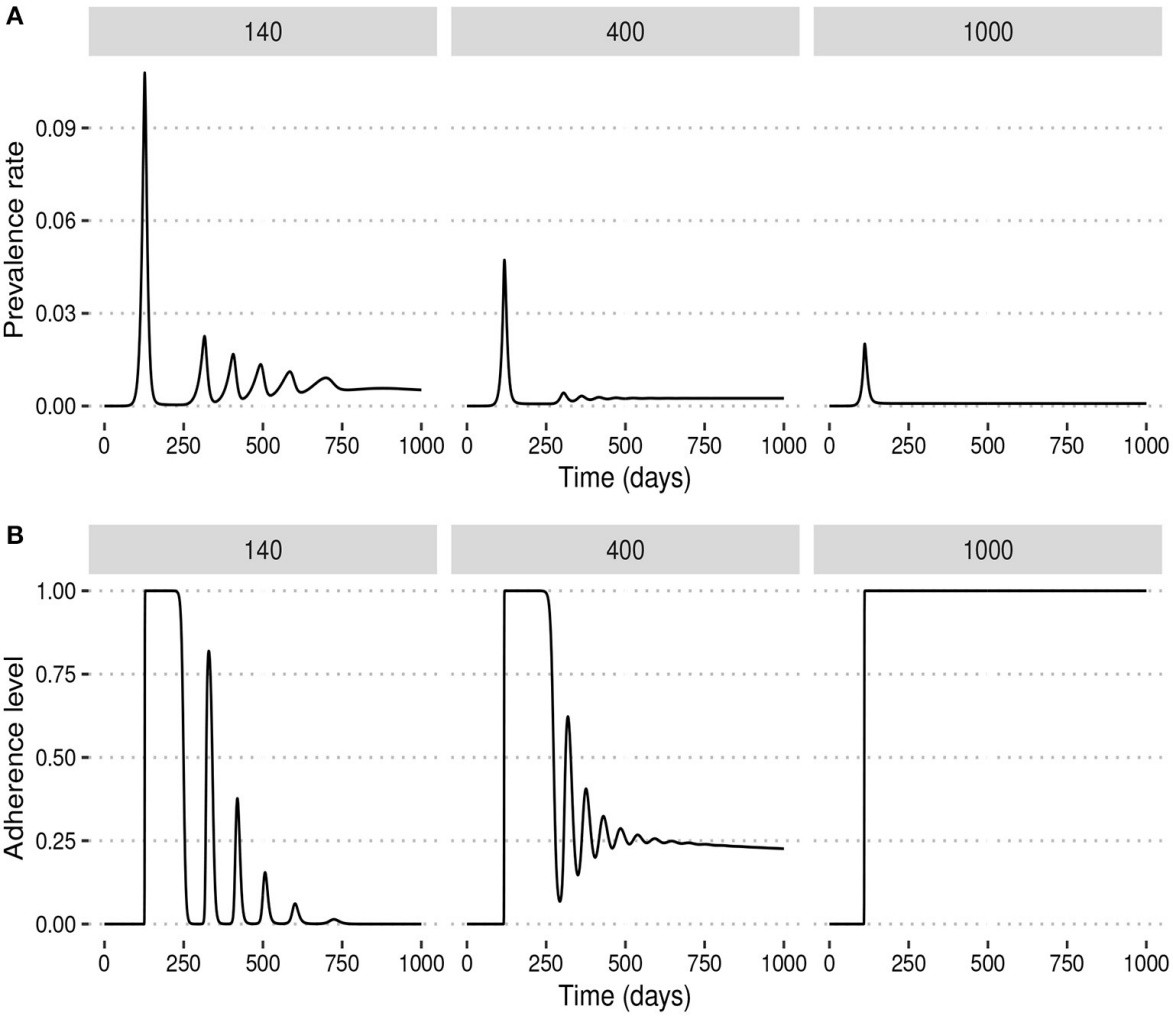
Behavior at equilibrium (endemic) for different values of the *ω*. For low values of *ω*, public tolerance for the disease is high and the system converges to a state where there is no adherence to NPIs during the endemic stage of the disease. For higher values of *ω*, however, NPI adherence during the endemic stage can be either partial or total depending on how large the value of *ω* is. **(A)** Prevalence rates. **(B)** Adherence levels.

Lastly, we simulate the modified SEIR model with a different set of parameter values to investigate the behavior of the system at equilibrium. In particular, we fix the NPI stringency at *α* = 2000 and use *ω* as the bifurcation parameter. Figure 6 shows that when *ω* is low, the NPI adherence level decays to zero as the disease becomes endemic. However, for larger values of *ω*, observe either partial or full adherence to NPIs at the endemic state depending on how large the value of *ω* is. Therefore, the system undergoes two transcritical bifurcations as the value of *ω* is varied. It is important to note that the exact values where these exchanges of stability occur depend on the values of other parameters. In the particular case of partial NPI adherence at equilibrium, increasing the value of *ω* results in both a decrease in the endemic prevalence rate and an increase in the NPI adherence level at equilibrium. Additionally, increasing the NPI stringency, *α*, at fixed *ω* can destabilize the system from full to partial adherence and even no adherence to NPIs at equilibrium.

## 4. Discussion

Non-pharmaceutical interventions play a central role in infectious disease outbreak response and control. These interventions can be very effective in curtailing the spread of infectious diseases when adequately implemented and sufficiently adopted by the population. However, NPIs can be very disruptive, and the socioeconomic and cultural challenges associated with their implementation interfere with both the ability and willingness of affected populations to adopt such interventions (25). This can lead to not only geographical but also temporal variations in adherence levels to NPIs during disease outbreaks and impede the control of infectious diseases (12). In this study, we investigated the interplay between NPI stringency, temporal variations in NPI adherence levels, and disease dynamics using mathematical modeling. We showed that when the NPI adherence level is assumed constant over time, a substantial increase in the stringency of NPIs would result in a substantial decrease in the peak prevalence and the epidemic curve can be flattened by imposing stringent disease control measures. The ultimate conclusion, in this case, is that the higher the stringency of NPIs, the more effective disease control is. Though this agrees with the results by Gros et al. (34), we showed that this conclusion might not hold when temporal variations in NPI adherence levels are taken into account. For time-varying adherence levels to NPIs, the dynamics of the disease are no longer influenced only by the stringency of control measures, but also by risk perception and the perceived frustration associated with NPIs. Our results suggest that when the public tolerance for the disease is relatively high, which might occur when either the perceived risk is low or the perceived frustration with NPIs is high, the level of adherence to NPIs might fluctuate over time, consistent with the results by Crane et al. (14). We showed that these fluctuations in NPI adherence levels over time might induce the occurrence of multiple epidemic waves, which is consistent with the results by Ochab et al. (37) and Ngonghala et al. (38). Furthermore, our results suggest a trade-off between NPI stringency and adherence in the sense that the public would find it more difficult to sustain high levels of NPI adherence when the stringency level is high, which is again consistent with the results by Acuña-Zegarra et al. (27). This conclusion is supported both by our scenario analysis and analytical derivations of the stability properties of the endemic equilibrium points of our model.

Our model also suggests that interventions that aim at increasing the adherence level to NPIs might be much more valuable than increasing the overall NPI stringency. In particular, we showed that an epidemic can be effectively controlled even with not very stringent NPIs if the ability and willingness of the public to adopt such interventions is sufficiently increased. We, therefore, argue that for the effective control of infectious disease outbreaks, public health authorities should not only focus on implementing the right policies but also, more importantly, on devising strategies to both increase risk perception and decrease the frustration associated with NPIs. This agrees with the conclusions by Avusuglo et al. (31). Importantly, our results suggest that much consideration should be given to the socioeconomic and cultural realities of affected populations when devising public health policies for infectious disease control.

Our work has important limitations that present opportunities for future studies. First, our model has not been calibrated to epidemic data. Also, we have kept the model simple for generality. However, to reflect real viral epidemics, it might be useful to take factors such as re-infections, vaccine availability, and the spread of multiple variants of the same virus into account. Furthermore, we assumed that the level of frustration with NPIs is constant over time. This might not be realistic. Accounting for temporal variations in frustration levels associated with NPIs might yield more insight. Lastly, we assumed that the implementation of NPIs was triggered by the prevalence of the disease. However, there might be cases where NPIs are implemented based on other factors such as the availability of intensive care units or the number of disease-induced deaths. It would be insightful to check whether the conclusions of this study are robust with respect to these other modeling approaches.

## Data availability statement

The original contributions presented in the study are included in the article/Supplementary material, further inquiries can be directed to the corresponding author/s.

## Author contributions

J-PN'k, CC, and FN: conception of the work, model formulation, critical revision of the important intellectual content, and provide approval for publication of the content. J-PN'k: model analysis and numerical simulations, drafting the work, and agreement to be accountable for all aspects of the work. CC and FN: supervision. All authors contributed to the article and approved the submitted version.
