# Supplementary material for: Effect of time-varying adherence to non-pharmaceutical interventions on the occurrence of multiple epidemic waves: A modeling study
Source: Front Public Health. 2022 Dec 20;10:1087683. doi: 10.3389/fpubh.2022.1087683 (PMC9807866; doi:10.3389/fpubh.2022.1087683)
Supplement: Supplementary file 1 [file Data_Sheet_1.pdf]

Supplementary Material

1 STABILITY PROPERTIES OF DISEASE-FREE EQUILIBRIA

THEOREM 1.1 (Local Stability). *The disease-free equilibrium (DFE) point $\mathcal{E}_1$ is locally asymptotically stable when $\mathcal{R}_0 < 1$, and unstable when $\mathcal{R}_0 > 1$. On the contrary, the DFE $\mathcal{E}_2$ is always unstable.*

PROOF. The jacobian matrix at a DFE is given by

$$\mathcal{J} = \begin{bmatrix} -\mu & 0 & -\beta_0 \frac{\pi}{\mu} & 0 & 0 \\ 0 & -(\kappa+\mu) & \beta_0 \frac{\pi}{\mu} & 0 & 0 \\ 0 & \kappa & -(\gamma+\delta+\mu) & 0 & 0 \\ 0 & 0 & \gamma & -\mu & 0 \\ 0 & 0 & 0 & 0 & \tau \end{bmatrix}$$

where $\tau = -K$ for the $\mathcal{E}_1$ and $\tau = K$ for the $\mathcal{E}_2$. The eigenvalues of $\mathcal{J}$ are given by

$$\begin{aligned} \lambda_1 &= \lambda_4 = -\mu \\ \lambda_2 &= -\frac{1}{2}(\gamma+\delta+\kappa+2\mu) + \frac{1}{2}\sqrt{(\delta+\gamma-\kappa)^2 + \frac{4\kappa\pi\beta_0}{\mu}} \\ \lambda_3 &= -\frac{1}{2}(\gamma+\delta+\kappa+2\mu) - \frac{1}{2}\sqrt{(\delta+\gamma-\kappa)^2 + \frac{4\kappa\pi\beta_0}{\mu}} \\ \lambda_5 &= \tau. \end{aligned}$$

For $\mathcal{E}_1$, only λ_2 can be positive. Hence, $\mathcal{E}_1$ is locally asymptotically stable if and only if $\lambda_2 < 0$. This is equivalent to

$$\begin{aligned} -(\gamma+\delta+\kappa+2\mu) + \sqrt{(\delta+\gamma-\kappa)^2 + \frac{4\kappa\pi\beta_0}{\mu}} &< 0, \\ (\delta+\gamma-\kappa)^2 + \frac{4\kappa\pi\beta_0}{\mu} &< (\gamma+\delta+\kappa+2\mu)^2 \\ \frac{4\kappa\pi\beta_0}{\mu} &< (\gamma+\delta+\kappa+2\mu)^2 - (\delta+\gamma-\kappa)^2 \\ \frac{\kappa\pi\beta_0}{\mu} &< (\gamma+\delta+\mu)(\kappa+\mu) = \chi \\ \mathcal{R}_0 &< 1. \end{aligned}$$

This last condition also holds for $\mathcal{E}_2$, but now $\tau = K > 0$. Therefore, $\mathcal{E}_2$ is always unstable.

THEOREM 1.2 (Global Stability of $\mathcal{E}_1$). *The DFE $\mathcal{E}_1$ is globally asymptotically stable when $\mathcal{R}_0 < 1$.*

We will use the following result by Chavez et al. (2002).

THEOREM 1.3. *Consider the epidemiological model:*

$$\begin{aligned} \frac{d\mathbf{x}}{dt} &= F(\mathbf{x}, \mathbf{I}), \\ \frac{d\mathbf{I}}{dt} &= G(\mathbf{x}, \mathbf{I}), \quad G(\mathbf{x}, 0) = 0, \end{aligned} \tag{S1}$$

where $\mathbf{x} \in \mathbb{R}^m$ denotes the number of uninfected individuals and $\mathbf{I} \in \mathbb{R}^n$ denotes the number of infected individuals including latent, infectious, etc. Let $\mathbf{U}_0 = (\mathbf{x}^*, \mathbf{0})$ denote the disease-free equilibrium of this system. Then $\mathbf{U}_0$ is a globally asymptotically stable equilibrium of the system (S1) provided $\mathcal{R}_0 < 1$ and the following assumptions (H1) *and* (H2) *are satisfied.*

(H1) *For $\frac{d\mathbf{x}^*}{dt} = F(\mathbf{x}, \mathbf{0})$, $\mathbf{x}^*$ is globally asymptotically stable,*

(H2) $G(\mathbf{x}, \mathbf{I}) = A\mathbf{I} - \widehat{G}(\mathbf{x}, \mathbf{I})$, $\widehat{G}(\mathbf{x}, \mathbf{I}) \geq 0$ *for* $(\mathbf{x}, \mathbf{I}) \in \Omega$, *where $A = D_{\mathbf{I}}G(\mathbf{x}^*, \mathbf{0})$ is an M-matrix (the off-diagonal elements of A are nonnegative) and Ω is the region where the model makes biological sense.*

PROOF. (of Theorem 1.2). For the purposes of this proof, we consider the adherence level x as one of the disease states. This artefact is crucial if we ought to apply Theorem 1.3. In that case, $\mathbf{x} = (S, R, x)$ and $\mathbf{I} = (E, I)$. Hence, the system $\frac{d\mathbf{x}^*}{dt} = F(\mathbf{x}, \mathbf{0})$ can be written as

$$\begin{aligned} S' &= \pi - \mu S, \\ R' &= -\mu R, \\ x' &= -Kx(1 - x). \end{aligned} \tag{S2}$$

This is an uncoupled system and it is easy to see that $\mathbf{x}^* = \left(\frac{\pi}{\mu}, 0, 0\right)$ is a globally asymptotically stable equilibrium of the system (S2). Moreover, for the modified SEIR model of interest,

$$A = \begin{pmatrix} -(\kappa + \mu) & \frac{\beta_0 \pi}{\mu} \\ \kappa & -(\gamma + \delta + \mu) \end{pmatrix},$$

and

$$\widehat{G}(\mathbf{x}, \mathbf{I}) = \beta_0 I \begin{pmatrix} \frac{\pi}{\mu} - \frac{S}{1+\alpha I x} \\ 0 \end{pmatrix}.$$

$\widehat{G}(\mathbf{x}, \mathbf{I}) \geq 0$ is equivalent to

$$\frac{\pi}{\mu} - \frac{S}{1 + \alpha I x} \geq 0,$$

and it is easy to see that this is satisfied in the feasible region Ω defined by

$$\Omega = \left\{ (S, E, I, R, x) \in \mathbb{R}_+^4 \times \mathbb{R}_+ : \; N \leq \frac{\pi}{\mu} \text{ and } x \leq 1 \right\}.$$

Based on Theorem (1.3), we conclude that $\mathcal{E}_1$ is globally asymptotically stable when $\mathcal{R}_0 < 1$.

2 STABILITY PROPERTIES OF ENDEMIC EQUILIBRIA

The jacobian matrix at an endemic equilibrium point $(S^*, E^*, I^*, R^*, x^*)$ is given by

$$\mathcal{J} = \begin{bmatrix} -\mu - \frac{\beta_0 I^*}{1+\alpha I^* x^*} & 0 & -\frac{\beta_0 S^*}{(1+\alpha I^* x^*)^2} & 0 & \frac{\alpha\beta_0 {I^*}^2 S^{*2}}{(1+\alpha I^* x^*)^2} \\ \frac{\beta_0 I^*}{1+\alpha I^* x^*} & -\kappa - \mu & \frac{\beta_0 S^*}{(1+\alpha I^* x^*)^2} & 0 & -\frac{\alpha\beta_0 {I^*}^2 S^{*2}}{(1+\alpha I^* x^*)^2} \\ 0 & \kappa & -(\delta+\gamma+\mu) & 0 & 0 \\ 0 & 0 & \gamma & -\mu & 0 \\ 0 & 0 & K\omega x^*(1-x^*) & 0 & K(1-2x^*)(-1+\omega I^*) \end{bmatrix}.$$

The characteristic polynomial can be written as

$$P(\lambda) = -\frac{\lambda+\mu}{1+\alpha x^* I^*} P'(\lambda) \tag{S3}$$

where

$$P'(\lambda) = K\kappa\alpha\beta_0\omega x^*(1-x^*)S^* {I^*}^2(\lambda+\mu) + \Bigg\{ \beta_0\kappa S^*(\lambda+\mu) \quad - \\ (1+\alpha x^* I^*)(\delta+\gamma+\mu+\lambda)(\kappa+\mu+\lambda)\Big[\lambda+\mu+\Big(\beta_0+\alpha x^*(\lambda+\mu)\Big)I^*\Big]\Bigg\}\times \\ \Big[-\lambda+K(1-2x^*)(-1+\omega I^*)\Big] \tag{S4}$$

THEOREM 2.1 (Stability of $\mathcal{E}_3$). *The endemic equilibrium point $\mathcal{E}_3$ is locally asymptotically stable when*

$$1 < \mathcal{R}_0 < 1 + \frac{\beta_0}{\mu\omega}, \tag{S5}$$

and unstable otherwise.

PROOF. Taking $x^* = 0$ into (S4) yields

$$P'(\lambda) = [\lambda - K(-1+\omega I^*)]P''(\lambda),$$

where $P''(\lambda) = \lambda^3 + a_1\lambda^2 + a_2\lambda + a_3$ and

$$a_1 = \xi + \mu + \beta_0 I^*, \tag{S6}$$

$$a_2 = \chi + \xi(\mu + \beta_0 I^*) - \beta_0\kappa S^*, \tag{S7}$$

$$a_3 = (\mu + \beta_0 I^*)\chi - \beta_0\kappa\mu S^*, \tag{S8}$$

with $\chi = (\gamma+\delta+\mu)(\kappa+\mu)$ and $\xi = \gamma + +\delta + \kappa + 2\mu$.

The Routh-Hurwitz criteria provide necessary and sufficient conditions for the roots of a polynomial to all have negative real parts (Brauer et al., 2019). For the polynomial of degree three $P''(\lambda)$, these conditions are

$$a_1 > 0 \text{ and } a_1 a_2 > a_3 > 0. \tag{S9}$$

- From (S6), it is obvious that $a_1 > 0$.
- Evaluating Eq. (S8) at $\mathcal{E}_3$ yields

$$a_3 = (\mathcal{R}_0 - 1)\mu\chi, \tag{S10}$$

  which is positive when $R_0 > 1$.
- Similarly, at

$$\mathcal{E}_3$$

  , Eq. (S6) & S7 yield $a_1 = \xi + \mu\mathcal{R}_0$ and $a_2 = \mu\xi\mathcal{R}_0$. Thus

$$a_1 a_2 - a_3 = (\xi^2 - \chi)\mathcal{R}_0 + \mu\xi\mathcal{R}_0^2 + \chi > 0$$

  as $\xi^2 - \chi = (\gamma + \delta + \mu)^2 + \chi + (\kappa + \mu)^2 > 0$.

These results show that the roots of $P''(\lambda)$ have negative real parts whenever the endemic point $\mathcal{E}_4$ exits, i.e. $\mathcal{R}_0 > 1$. Therefore, $\mathcal{E}_4$ is locally asymptotically stable if the remaining eigenvalue

$$\lambda = K(-1 + \omega I^*)$$

is negative. It is easy to see that this is equivalent to $\mathcal{R}_0 < 1 + \frac{\beta_0}{\mu\omega}$ at $\mathcal{E}_3$.

THEOREM 2.2 (Stability of $\mathcal{E}_5$). *The endemic equilibrium point $\mathcal{E}_5$ is locally asymptotically stable when*

$$\mathcal{R}_0 > 1 + \frac{\beta_0}{\mu\omega} + \frac{\alpha}{\omega} \quad \text{(S11)} \qquad \text{and} \qquad \mathcal{R}_0 > \frac{1}{\mu}\left(\frac{\chi}{\xi_0} - 1\right), \quad \text{(S12)}$$

where $\chi = (\gamma+\delta+\mu)(\kappa+\mu)$ and $\xi_0 = \gamma+\delta+\kappa+2\mu$. If the epidemiologically plausible assumption $\kappa \leq 1$ is satisfied, then (S11) subsumes (S12). The endemic equilibrium $\mathcal{E}_5$ is unstable when $\mathcal{R}_0 < 1 + \frac{\beta_0}{\mu\omega} + \frac{\alpha}{\omega}$.

PROOF. Proceeding as in the proof of Theorem 2.1, we evaluate $P'(\lambda)$ at $x^* = 1$ to obtain

$$P'(\lambda) = -\big[\lambda + K(-1 + \omega I^*)\big]P''(\lambda),$$

where $P''(\lambda) = \lambda^3 + b_1\lambda^2 + b_2\lambda + b_3$ and

$$b_1 = \frac{1}{1 + \alpha I^*}\Big[\xi_1(1 + \alpha I^*) + \beta_0 I^*\Big], \tag{S13}$$

$$b_2 = \frac{1}{(1 + \alpha I^*)^2}\Big[(1 + \alpha I^*)\Big(\xi_2 + (\beta_0\xi_0 + \alpha\xi_2)I^*\Big) - \beta_0\kappa S^*\Big], \tag{S14}$$

$$b_3 = \frac{1}{(1 + \alpha I^*)^2}\Big[\chi(1 + \alpha I^*)\Big(\mu + (\beta_0 + \alpha\mu)I^*\Big) - \beta_0\mu\kappa S^*\Big], \tag{S15}$$

with $\xi_0 = \gamma + \delta + \kappa + 2\mu$, $\xi_1 = \xi_0 + \mu$ and $\xi_2 = \chi + \mu\xi_0$.

1. Routh-Hurwitz Conditions
   - b_1 is always positive.
   - Evaluating Eq. (S15) at $\mathcal{E}_5$ we obtain

$$b_3 = \frac{\mu\chi}{\beta_0 + \alpha\mu}\Big[\alpha\mu\mathcal{R}_0(\mathcal{R}_0 - 1) + (\beta_0\mathcal{R}_0 - 1)\Big],$$

   which is positive if $\mathcal{R}_0 > 1$ and $\mathcal{R}_0 > \frac{1}{\beta_0}$.
   - Let $c_1 = b_1 b_2 - b_3$. Then

$$c_1 = \frac{\mu(\beta_0 + \alpha\mu\mathcal{R}_0)}{\beta_0 + \alpha\mu)^4} c_2,$$

   where

$$c_2 = -\chi(\beta_0 + \alpha\mu)^3(\mathcal{R}_0 - 1) + (\beta_0 + \alpha\mu\mathcal{R}_0)\Big[(\beta_0 + \alpha\mu\mathcal{R}_0) + \mu(\beta_0 + \alpha\mu)\mathcal{R}_0\Big]\times \Big[\alpha\chi(\mathcal{R}_0 - 1) + (\beta_0 + \alpha\mu)\xi_0\mathcal{R}_0\Big]. \quad \text{(S16)}$$

   When $\mathcal{R}_0 > 1$, $\beta_0 + \alpha\mu\mathcal{R}_0 > \beta_0 + \alpha\mu$ and (S16) yields

$$\begin{aligned} c_2 &> (\beta_0 + \alpha\mu)^2 c_3, \\ c_3 &= -\chi(\beta_0 + \alpha\mu)(\mathcal{R}_0 - 1) + (1 + \mu\mathcal{R}_0)\Big[\alpha\chi(\mathcal{R}_0 - 1) + (\beta_0 + \alpha\mu)\xi_0\mathcal{R}_0\Big], \\ &= (\beta_0 + \alpha\mu)\mathcal{R}_0\Big[\xi_0(1 + \mu\mathcal{R}_0) - \chi\Big] + \chi(\beta_0 + \alpha\mu) + \alpha\chi(\mathcal{R}_0 - 1)(1 + \mu\mathcal{R}_0). \end{aligned}$$

   A sufficient condition for $c_1 > 0$ when $\mathcal{R}_0 > 1$ is therefore $\xi_0(1 + \mu\mathcal{R}_0) > \chi$. If we assume $\kappa \leq 1$, then

$$\begin{aligned} \xi_0(1 + \mu\mathcal{R}_0) - \chi &> \xi_0(\kappa + \mu) - \chi \quad \text{when } \mathcal{R}_0 > 1, \\ &> \big[(\gamma + \delta + \mu) + (\kappa + \mu)\big](\kappa + \mu) - \chi, \\ &> (\kappa + \mu)^2, \end{aligned}$$

   which means $c_1 > 0$ whenever $\mathcal{R}_0 > 1$ and $\kappa \leq 1$.
2. The remaining eigenvalue $\lambda = -K(-1 + \omega I^*)$ is negative if $I^* > 1/\omega$. At $\mathcal{E}_5$, this is equivalent to

$$\mathcal{R}_0 > 1 + \frac{\beta_0}{\mu\omega} + \frac{\alpha}{\omega}.$$

REFERENCES

Brauer, F., Castillo-Chavez, C., and Feng, Z. (2019). *Mathematical Models in Epidemiology*, vol. 32 (Springer)

Chavez, C. C., Feng, Z., and Huang, W. (2002). On the computation of r0 and its role on global stability. *Mathematical Approaches for Emerging and Re-emerging Infection Diseases: An Introduction* 125, 31–65
